# Global research on the crosstalk between microbiota - intratumoral microorganisms and liver cancer: a visualization analysis

**DOI:** 10.1186/s13027-026-00750-x

**Published:** 2026-04-20

**Authors:** Huaxiang Jiang, Yejing Cui, Junqiang Lei

**Affiliations:** 1 The First Clinical Medical College of Lanzhou University, Lanzhou, China; 2 Gansu Province Clinical Research Center for Radiology Imaging, Lanzhou, China; 3 Intelligent Imaging Medical Engineering Research Center of Gansu Province, Lanzhou, China; 4 Accurate Image Collaborative Innovation International Science and Technology Cooperation Base of Gansu Province, Lanzhou, China

**Keywords:** Intratumoral microbiota, Liver cancer, Bibliometric, Tumor microenvironment

## Abstract

**Background:**

In the past few decades, the field of microbiota research has experienced rapid development and growth. We have employed bibliometric methods to comprehensively visualize and analyze the global knowledge and hotspots in the field of microbiome-intratumoral microbiota in liver cancer.

**Method:**

The relevant literature in this field from 2009 to 2025 was extracted from the Web of Science Core Collection Database. After the data was extracted, it was analyzed and visualized using CiteSpace, VOSviewer and R (bibliometrix) software.

**Result:**

A total of 1001 publications on microbiome - intratumoral microbiota and liver cancer were published during the period 2009–2025. Among these, China had the highest number of publications (*n* = 495). The most prolific institution publishing on microbiome - intratumoral microbiota and liver cancer was Huazhong University of Science and Technology, China (*n* = 29). The author with the most publications on this topic was Yu, Jun (*n* = 14, 1.4%). The journal with the highest number of publications on this subject was Cancers (*n* = 41, 4.1%). The top seven keywords with a frequency of 100 or more include: gut microbiota, hepatocellular carcinoma, nonalcoholic fatty liver disease, fatty liver disease, cancer, inflammation, bile acids, liver cancer, cell, and insulin resistance. Recent emerging topics include “intratumoral microbiota” (since 2024) and “tumor microenvironment.”

**Conclusion:**

Current research in this field primarily investigates the mechanistic associations between gut microbiota and hepatic malignancies, with particular emphasis on hepatocellular carcinoma. The scientific frontier has progressively evolved to encompass the exploration of intratumoral microbiota and its multifaceted interactions within the tumor microenvironment.

**Supplementary Information:**

The online version contains supplementary material available at 10.1186/s13027-026-00750-x.

## Introduction

Liver cancer is particularly a malignant tumor with extremely high incidence and mortality rates worldwide [1]. Its development is a complex, multi-step, and multi-factorial process, involving the continuous progression of chronic liver diseases (such as viral hepatitis, alcoholic liver disease, and metabolic dysfunction-associated steatotic liver disease (MASLD)) [2]. In recent years, with the in-depth study of microbiomics, the role of microbiota in tumorigenesis has increasingly attracted attention. Initial research primarily focused on the indirect effects of gut microbiota on liver cancer through the “gut-liver axis” [3]. However, growing evidence suggests that tumor tissues also harbor their unique microbial communities, known as intratumoral microbiota [4]. These microorganisms colonized in the tumor microenvironment are believed to directly participate in the progression, metastasis, and treatment response of liver cancer through various mechanisms, such as regulating immune responses, influencing cell metabolism, and inducing gene mutations [5]. Although this emerging field shows great potential, the global research landscape, knowledge structure, and developmental trajectory still need to be systematically organized.

Bibliometrics is a discipline that employs mathematical and statistical methods to quantitatively analyze academic literature in specific fields [6]. It can intuitively reveal the developmental dynamics, knowledge structure, research hotspots, and frontier trends of a particular research area by parsing the intrinsic relationships within massive literature data [7]. Compared to traditional reviews, bibliometric analysis provides a more objective, macro, and systematic perspective, identifying core authors, key countries/regions, high-impact institutions, and core journals in the field, and mapping out scientific research collaboration networks [8]. Furthermore, through keyword co-occurrence and burst detection analysis, it can precisely locate research focuses and emerging trends in different periods, thereby offering robust data support for researchers to quickly grasp the overall landscape of the field, seek collaboration opportunities, and plan future research directions.

This study aims to comprehensively review and visually analyze global scientific research on the interaction between intratumoral microbiota and liver cancer using bibliometric methods. To systematically address the core question of “What is the current global research status, collaboration patterns, knowledge base, and evolutionary trends in this field?“, we adopted a multi-tool cross-validation analysis strategy. Specifically, we utilized VOSviewer (1.6.20) software [9] to conduct research collaboration network analysis of countries/regions and institutions, co-occurrence analysis of authors and journals, and constructed a keyword co-occurrence network to identify research topic clusters. Simultaneously, we employed CiteSpace (6.4.R1) software [10] for keyword time-zone view analysis and burst detection to capture the evolutionary paths of research hotspots and frontier mutation terms. Additionally, we used the bibliometrix package [11] in R software to draw country collaboration maps, perform core journal analysis, and generate keyword trend topic maps and trinity diagrams, revealing the knowledge flow structure from multiple dimensions.

The innovation and significance of this study lie in the following aspects: Firstly, it represents the first systematic bibliometric exploration in the cutting-edge interdisciplinary field of “intratumoral microbiota and liver cancer”. Secondly, we have comprehensively employed three complementary bibliometric tools, ensuring the comprehensiveness and robustness of the analysis results. This multi-platform integration strategy demonstrates distinct advantages in similar studies. Ultimately, this study aims to provide scholars, clinicians, and research policymakers with a clear “knowledge map” of the field, assisting them in understanding the current research landscape, identifying key breakthroughs, and anticipating future development directions, thereby promoting in-depth exploration and clinical translation in this field.

## Materials and methods

### Data collection and search strategy

**Fig. 1:**
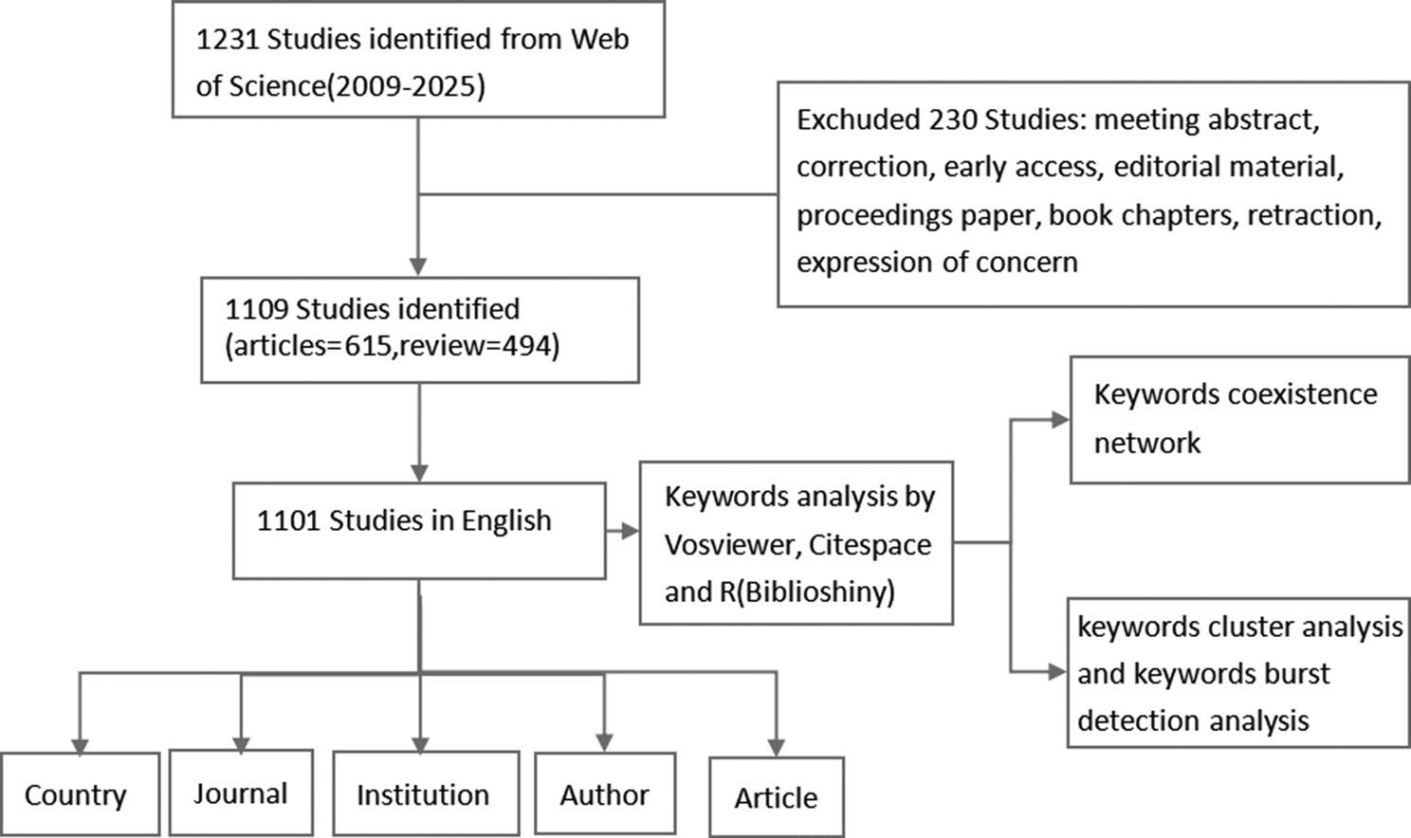
Detailed workflow

On October 13, 2025, we retrieved and downloaded data samples from the Web of Science Core Collection database. The retrieval rule was based on the TKA (Title + Author Keywords + Abstract) method [12]. The search strategy for the WoSCC database was formulated based on the retrieval rules, referencing the MeSH terms, entry terms, and subordinate terms from the PubMed database. The specific search strategy in the WoSCC database was ((TI=(microbiom* OR microbiota OR “intratumoral bacteri*” OR “tumor microbi*”)) OR (AB=(microbiom* OR microbiota OR “intratumoral bacteri*” OR “tumor microbi*”)) OR (AK=(microbiom* OR microbiota OR “intratumoral bacteri*” OR “tumor microbi*”))) AND ((TI=(“hepatocellular carcinoma” OR HCC OR “primary liver cancer” OR “cholangiocarcinoma” OR “intrahepatic cholangiocarcinoma” OR ICC OR “liver metastasis” OR “liver cancer” OR “hepatic metastasis” OR “liver metastases”)) OR (AB=(“hepatocellular carcinoma” OR HCC OR “primary liver cancer” OR “cholangiocarcinoma” OR “intrahepatic cholangiocarcinoma” OR ICC OR “liver metastasis” OR “liver cancer” OR “hepatic metastasis” OR “liver metastases”)) OR (AK=(“hepatocellular carcinoma” OR HCC OR “primary liver cancer” OR “cholangiocarcinoma” OR “intrahepatic cholangiocarcinoma” OR ICC OR “liver metastasis” OR “liver cancer” OR “hepatic metastasis” OR “liver metastases”))). The final publication date of the literature was limited to the period from January 1, 2009, to September 30, 2025, and the literature types were restricted to articles and reviews, with the language limited to English. To ensure the reliability and authenticity of the results, the literature screening was conducted by two independent professionals, excluding irrelevant literature. The specific operational process is illustrated in Fig. 1.

### Data analysis

In the WoSCC, we analyzed the annual publication count, top 10 institutions, and top 10 journals based on the number of retrieved publications. Subsequently, we employed VOSviewer, CiteSpace, R software, and Excel tools for data analysis [13]. Additionally, prior to analysis, we deduplicated countries, institutions, and keywords, with specific deduplication details available in the appendix file. These thresholds were not set randomly, they were established through a preliminary analysis of the dataset’s distribution, specifically by identifying the statistical inflection points. The retrieved literature included the legacy term “NAFLD” to maximize coverage, the results and discussion sections now consistently refer to the condition as “MASLD” in accordance with current guidelines.

VOSviewer is a software program developed by Leiden University in the Netherlands, used for creating, visualizing, and exploring maps based on network data [14]. We utilized VOSviewer (version 1.6.20) for literature analysis, including country/region and institutional research collaboration network analysis, author and journal analysis, keyword co-occurrence and clustering analysis [15].

CiteSpace is an information visualization software developed by Professor Chaomei Chen, an internationally renowned expert in information visualization, using Java language based on citation analysis theory [16]. We used CiteSpace (version 6.4.R1) for keyword co-occurrence clustering analysis and keyword burst detection analysis in the literature [17].

Systematic scientific mapping analysis was conducted using the “biblioshiny” web interface of the “bibliometrix” package in the R programming language [18], including: country collaboration map, core journal analysis, and Trend topics and Three-fields plot analysis of keywords.

## Result

### Annual publication trend

**Fig. 2:**
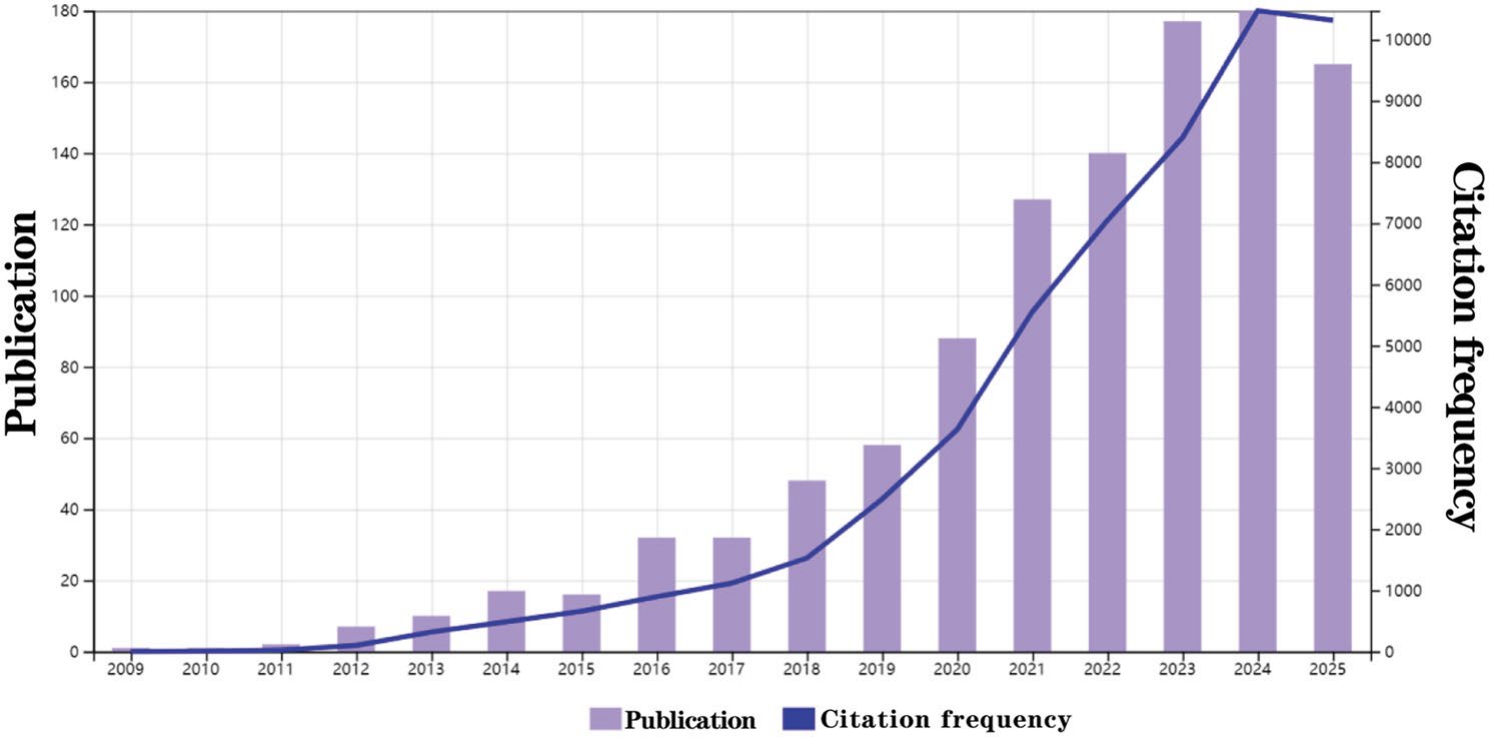
Contribution characteristics of the crosstalk between microbiota - intratumoral microorganisms and liver cancer in SCI research. Annual publication volume and citation frequency of global crosstalk between microbiota - intratumoral microorganisms and liver cancer and SCI studies

In the years 2009 and 2010, only one research article was published each year. Starting from 2011, there has been a gradual increase in attention towards this field of study, with the number of articles published annually rising steadily. By 2024, 180 related research articles were published, and as of October 2025, a total of 1101 studies have been published in SCI research, with the trend still on the rise. The citation frequency has also been increasing year by year from 2009 to 2025 (Fig. 2).

### The co-authorship country/region and institution cooperation network

**Fig. 3:**
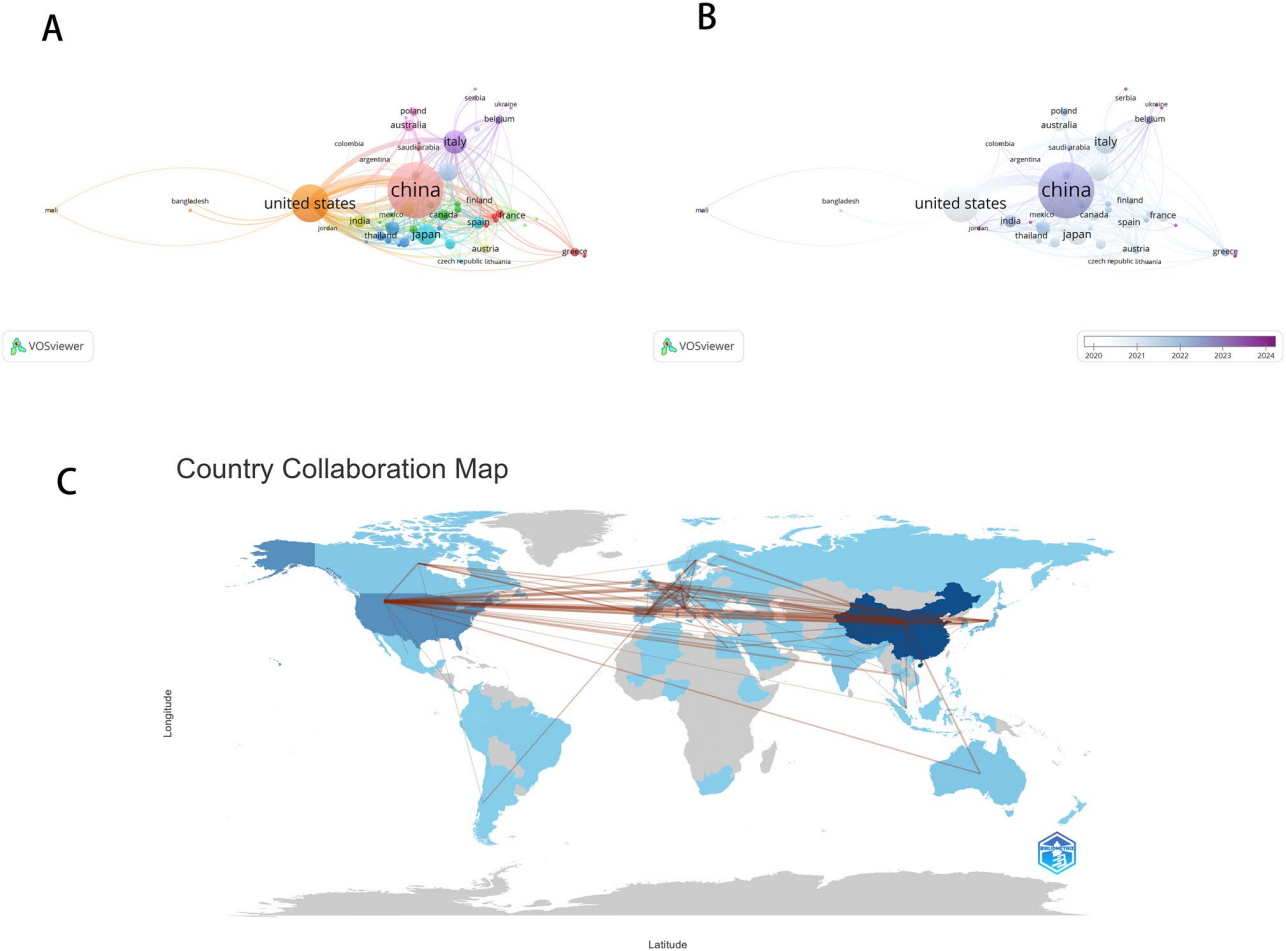
The co-authorship cooperation network of countries/regions created with VOSviewer(3**A**/**B**). The country collaboration map created with R (bibliometrix) (3**C**)

As shown in Fig. 3A, research topics related to the relationship between intratumoral microbiota and liver cancer are primarily concentrated in China and the United States on a global scale. China maintains close connections with various countries/regions, occupying a pivotal position. China has the highest number of publications (*n* = 495), followed by the United States (*n* = 236) and Italy (*n* = 97). However, the United States has the highest number of citations (*n* = 22,143), while China ranks second (*n* = 17,428), suggesting that Chinese publications still lag behind in terms of overall academic impact or visibility. The timeline curve in Fig. 3B reveals that China has been the most active and prolific country in recent years, and its significant role cannot be overlooked.

Figure 3C illustrates the co-authorship relationships among contributing countries in the studied literature, highlighting the most frequent collaborative links (with an absolute frequency ≥ 5 as the reference threshold). The lines in the figure represent the strength of collaboration between countries, with the thickness of the lines reflecting the frequency of collaboration. The strongest collaborative link is between China and Japan, with a frequency of 138.03, indicating a very close academic collaboration. The collaboration between China and South Korea ranks second with a frequency of 127.84, while the collaboration between China and Australia, with a frequency of 134.49, also represents a high-intensity collaboration. This collaboration network clearly reflects the main directions and intensity of global academic collaboration. China occupies a central position in the collaboration network, maintaining high-intensity collaborations with multiple Asian countries as well as Australia and the United States (see Appendix Table 1).

The top ten most prolific institutions, each having published at least 19 papers on microbiota and liver cancer research, include 8 from China and 2 from the United States (see Table 1). Among these ten institutions, four primarily focus on publishing research in the WoS category “Materials Science Multidisciplinary” during the period 2009–2025, and all four are from China. Three of the ten institutions mainly publish research in the WoS category “Oncology”. The most prolific institution, Huazhong University of Science and Technology in China, primarily publishes in the WoS category “Materials Science Multidisciplinary.”

**Table 1:** Top ten most prolific institutions publishing on microbiome - intra-tumor microbes and liver cancer, and the main WoS category from 2009 to 2025

Name of Institution, Country(Number of publications)	Main WoS Category(Number of publications)
Huazhong University of Science and Technology, China(*n* = 29)	Materials Science Multidisciplinary(*n* = 14686)
National Cancer Institute, USA(*n* = 28)	Oncology(*n* = 120)
Sun Yat-sen University, China(*n* = 28)	Oncology(*n* = 13815)
Fudan University, China(*n* = 26)	Oncology(*n* = 12660)
Zhejiang University, China(*n* = 24)	Materials Science Multidisciplinary(*n* = 19466)
The Chinese University of Hong Kong, China(*n* = 23)	Engineering Electrical Electronic(*n* = 4886)
Chinese Academy of Sciences, China(*n* = 20)	Materials Science Multidisciplinary(*n* = 119842)
Shanghai Jiao Tong University, China(*n* = 19)	Materials Science Multidisciplinary(*n* = 19695)
Shanghai University of traditional Chinese Medicine, China(*n* = 19)	Pharmacology Pharmacy(*n* = 3404)
University of California, San Diego, USA(*n* = 19)	Multidisciplinary Sciences(*n* = 8218)

### The author and journal analysis

**Fig. 4:**
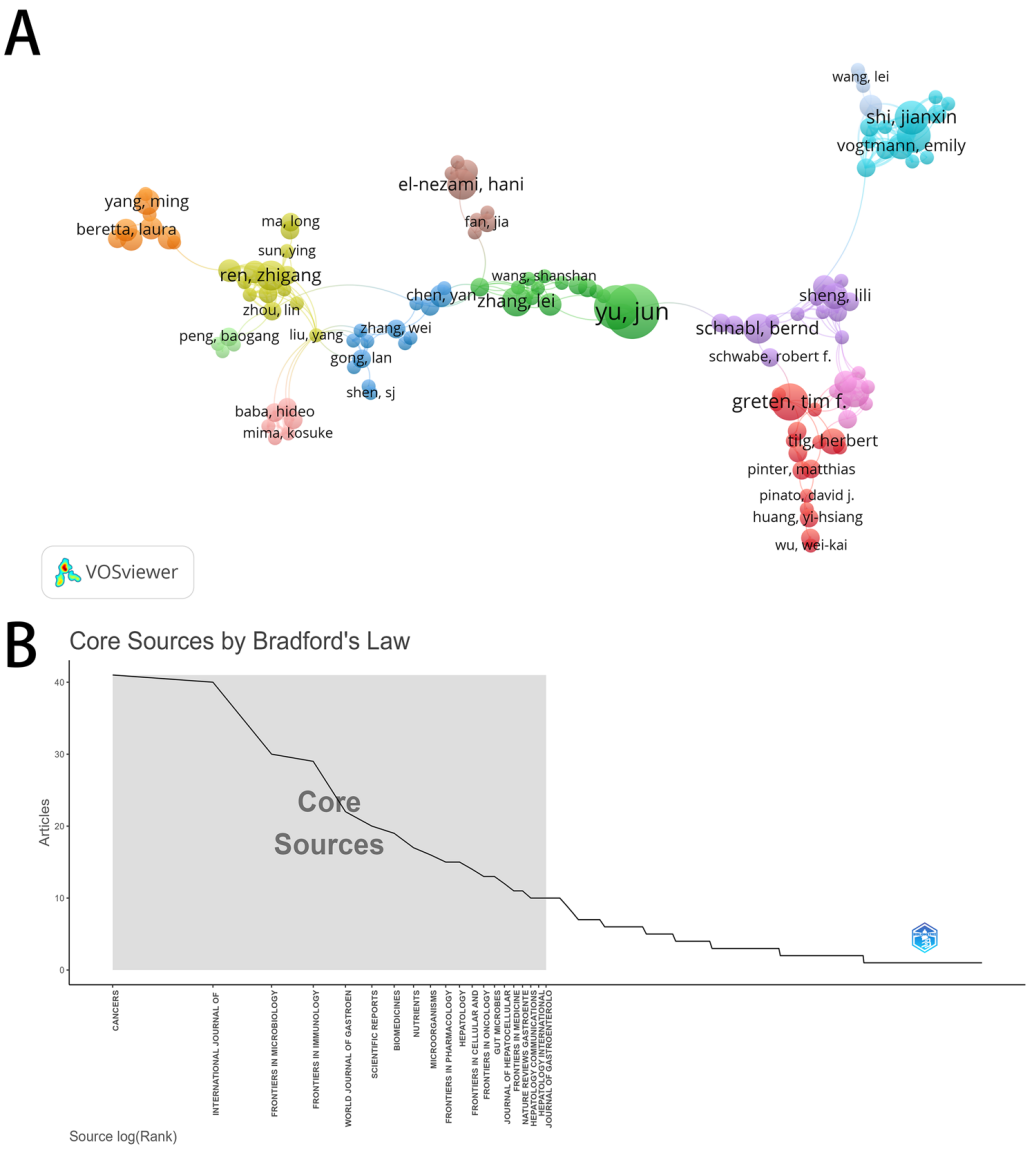
Top 265 most prolific co-authors in 2009–2025 and their relationships within 10 clusters by different colors, created by VOSviewer(4**A**). 4**B** was core sources by Bradford’s Law

During the period from 2009 to 2025, a total of 6,878 authors were identified in the published relevant literature. Among them, 265 authors were classified as the most prolific authors, each having published at least three or more related articles. Their relationship network analysis within 10 clusters, differentiated by colors, was created using VOSviewer (see Fig. 4A). The author with the highest number of published related articles is Yu Jun (*n* = 14, 1.4%), followed by Wang Yi (*n* = 13, 1.3%) (see Table 2).

Figure 4B presents the journal dispersion analysis based on Bradford’s Law, indicating that the literature in this field is highly concentrated in a few core journals. The core zone (Zone 1) includes only 20 journals (4.9% of the total number of journals), yet it contributes 368 articles (33.4% of the total number of articles). “Cancers”,“International Journal of Molecular Sciences” and “Frontiers in Microbiology” are the most representative journals with concentrated publication volumes among them. The intermediate zone (Zone 2) and the peripheral zone (Zone 3) cover 80 and 307 journals, respectively, with their literature contributions tending to be more dispersed (see Appendix Table 2). This distribution pattern strongly validates Bradford’s Law, which posits that the majority of important papers in a discipline are published in a few core journals.

Research on the microbiome-intratumoral microbes and liver cancer was published in 407 journals from 2009 to 2025 (see Table 3). The journal with the highest output on this topic is “Cancers” (*n* = 41, 4.1%), followed by “International Journal of Molecular Sciences” (*n* = 40, 4.0%), “Frontiers in Microbiology” (*n* = 30, 3.0%) and “Frontiers in Immunology” (*n* = 29, 2.9%).

**Table 2:** Top 10 most prolific authors on on microbiome - intra-tumor microbes and liver cancer

Authors (Country)	The number of documents	The number of citations
Yu, Jun (China)	14	1024
Wang, Yi (China)	13	315
Gasbarrini, Antonio (Italy)	12	844
Chen, Gang (China)	11	266
Zhang, Xiang (America)	11	1013
Ponziani, Francesca romana (Italy)	10	830
Greten, Tim f. (Germany)	9	1944
Abenavoli, Ludovico (Italy)	8	203
Bo, Zhiyuan (China)	8	163
Honda, Takashi (Japan)	8	109

**Table 3:** Top 10 most prolific journals on on microbiome - intra-tumor microbes and liver cancer

Journal, Publisher	Number of publications,%
Cancers, MDPI	41, 4.1%
International Journal of Molecular Sciences, MDPI	40, 4.0%
Frontiers in Microbiology, Frontiers Media SA	30, 3.0%
Frontiers in Immunology, Frontiers Media SA	29, 2.9%
World Journal of Gastroenterology, Baishideng Publishing Group	22, 2.2%
Scientific Reports, Springer Nature	20, 2.0%
Biomedicines, MDPI	19, 1.9%
Nutrients, MDPI	17, 1.7%
Microorganisms, MDPI	16, 1.6%
Frontiers in Pharmacology, Frontiers Media SA	15, 1.5%

### Keyword analysis

#### Co-occurrence all keywords cooperation network and trend topics

**Fig. 5:**
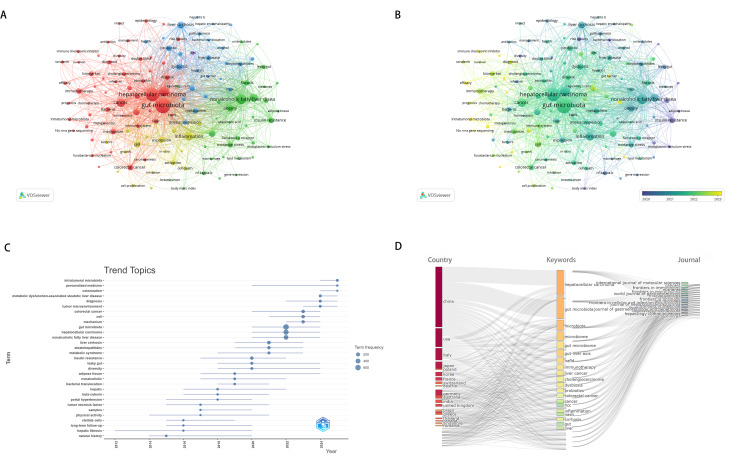
Top 129 most prolific Keywords on the microbiome and liver cancer and Keywords co-occurrence relationship network by different colors, created by VOSviewer(5**A**/**B**). Trend topics in the study domain of on the crosstalk between microbiota - intratumoral microbiota and liver cancer(5**C**). Three-fields plot linking keywords with the relevant contributing countries and journals in the studied field(5**D**)

Figure 5A illustrates the most prolific keywords on the microbiome and liver cancer and keywords co-occurrence relationship network by different colors, created by VOSviewer. The minimum number of occurrences of a keyword was set to 15, there were 129 keywords meeting the threshold. The 129 keywords of the articles published in 2009–2025 revealed five clusters, which can be categorized as follows: (1) the relationship between microbiota and cancer (red, 53 keywords); (2) metabolic liver diseases and lipid metabolism regulation (green, 33 keywords); (3) the gut-liver axis and the progression of chronic liver diseases (blue, 25 keywords); (4) molecular mechanisms of liver diseases and cellular regulatory networks (yellow, 17 keywords); (5) diet (purple, 1 keyword). Figure 5B demonstrates that the top 129 most prolific keywords on the microbiome and liver cancer represent the latest research areas in recent years.

To demonstrate the trending topics in the field of microbiome and liver cancer research, keywords that appeared at least five times were considered. The most frequently occurring keywords each year during the study period (2009–2025) are shown in Fig. 5C. Since no keywords appeared at least five times between 2009 and 2012, the time range displayed in the figure starts from 2012. From a temporal distribution perspective, the research trends exhibit a clear transition from fundamental mechanisms to clinical translation and cutting-edge subfields. In the early stage (2014–2018), the focus was on basic pathological mechanisms and cellular processes such as “liver fibrosis”, “tumor necrosis factor” and “β-catenin”. In the mid-term (2019–2021), attention gradually shifted to mechanisms of metabolic diseases and host-microbiota interactions, including “insulin resistance”,“gut dysbiosis” and “metabolic syndrome”. In the recent period (2022–2025), research hotspots have further transitioned to more clinically and precision medicine-oriented directions, such as “tumor microenvironment”,“intratumoral microbiota”,“metabolic dysfunction-associated steatotic liver disease” and “personalized medicine”. Notably, emerging themes include the integration of “intratumoral microbiota” (starting in 2024) with the “tumor microenvironment”, reflecting new insights into the role of microbes in tumor immunity and treatment response.

Based on the provided Three-Field Plot (Fig. 5D), the association structure among high-frequency keywords, core research countries, and publishing journals in this field was analyzed. The left side of the figure displays the main research countries, the middle section shows the top 20 high-frequency keywords, and the right side presents representative journals. In terms of countries, China, the United States, Italy, and Japan are the primary contributors. It is noteworthy that the United States is more focused on keywords such as “microbiome”,“liver cancer” and “immunotherapy”, indicating its leading position in these areas. China follows closely in research volume, particularly in fields related to “gut microbiome”,“hepatocellular carcinoma” and “colorectal cancer”. Italy ranks third, with its research emphasis on clinical and microecological interventions such as “dysbiosis”,“probiotics” and “liver cirrhosis”. Meanwhile, journal publications exhibit a multi-level distribution from basic molecular mechanisms (e.g., International Journal of Molecular Sciences) to immune microenvironments (e.g., Frontiers in Immunology) and classical hepatology (e.g., World Journal of Gastroenterology), reflecting the high correlation between research content and publishing platforms in this field.

#### Keywords network analysis, keywords cluster analysis and keywords burst detection analysis

**Fig. 6:**
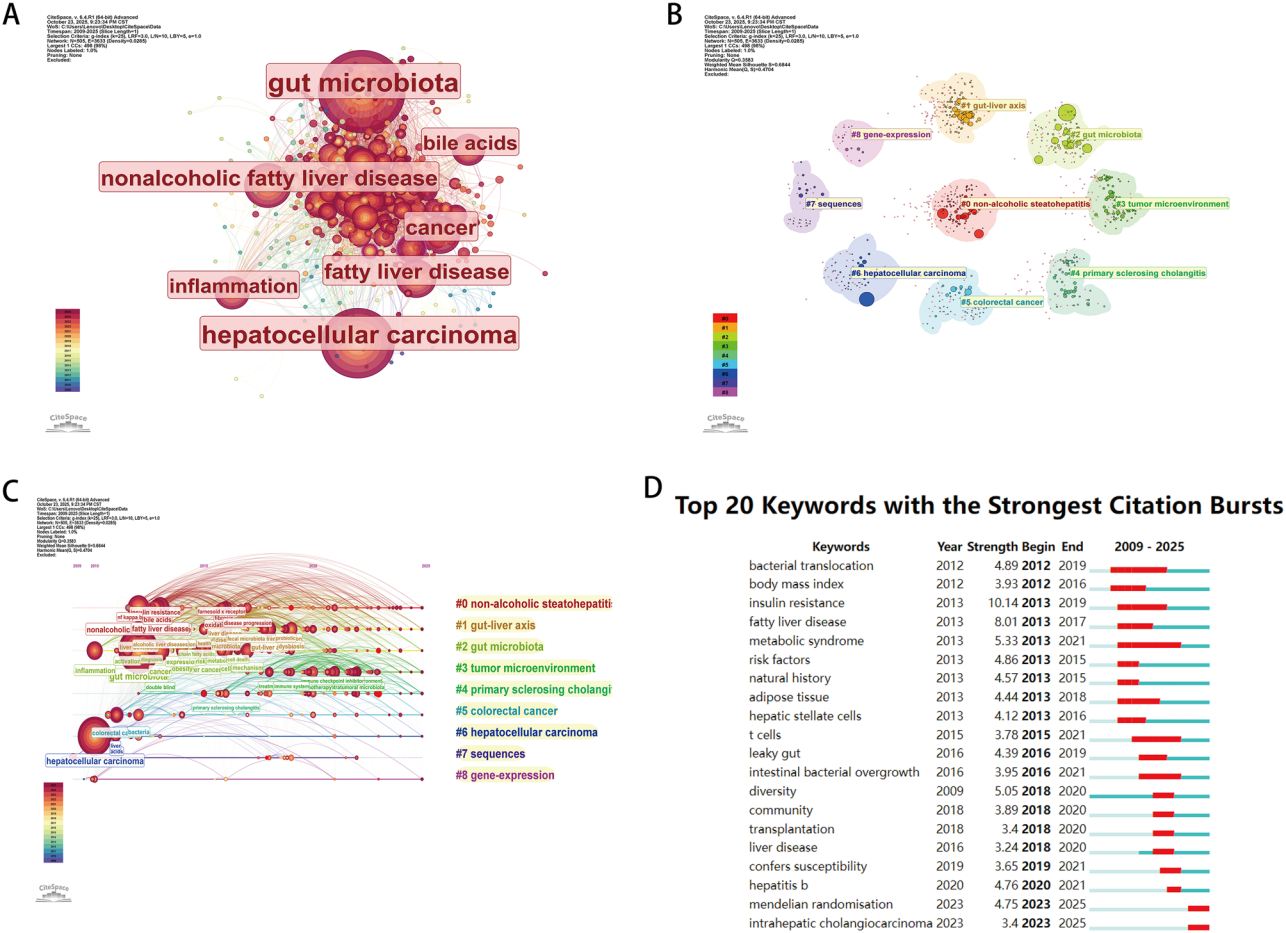
Keyword network analysis (**A**), Keyword cluster analysis (**B**/**C**), Keyword burst detection analysis (**D**), created by the CiteSpace software

Keyword co-occurrence can effectively reflect research hotspots in the field of microbiome-intratumoral microorganisms and liver cancer, while burst keywords can indicate frontier topics. To explore the distribution of research hotspots, frontier topics, and keywords, the first step is to analyze the co-occurrence network of keywords. Figure 6 nodes represent corresponding keywords, and the size of the nodes indicates the number of articles containing the keywords in this domain. The connecting lines between nodes represent the relationships between keywords. As shown in Fig. 6A, the connecting lines between various keywords are intricate, indicating complex interconnections. The top seven keywords with a frequency of occurrence equal to or greater than 100 times include: gut microbiota, hepatocellular carcinoma, nonalcoholic fatty liver disease, fatty liver disease, cancer, inflammation, bile acids, liver cancer, cell and insulin resistance. Among these, gut microbiota has the largest node and the most complex network of connecting lines.

We hierarchically arranged the keywords from all articles in the field of microbiome-intratumoral microbiota and liver cancer (2009–2025) through a clustering network. Figure 6B shows that different co-occurring keywords are represented by nodes, and different disciplines or topics are represented by clustering collectives. The co-occurrence keyword knowledge graph reveals keywords with high centrality and frequency of occurrence. The area of each node is proportional to the total literature frequency of the related research topic. Furthermore, Fig. 6C reveals that the distribution of high-frequency keywords in each subcluster along the timeline from 2009 to 2025. The co-occurrence keywords can be divided into 9 subclusters, including: #0 non-alcoholic steatohepatitis, #1 gut-liver axis, #2 gut microbiota, #3 tumor microenvironment, #4 primary sclerosing cholangitis, #5 colorectal cancer, #6 hepatocellular carcinoma, #7 sequences, #8 gene-expression. The recently emerging topic keywords include: “intratumoral microbiota” and “tumor microenvironment”.

The so-called “burst terms” refer to words that are frequently cited within a certain period. CiteSpace is used to detect burst keywords, which are considered indicators of research frontier topics. The timeline is represented by a blue line, and the time interval of keyword bursts is indicated by the red segment on the blue timeline. It represents the start year, end year, and duration of the research (Fig. 6D). The top 10 keywords in terms of duration include: metabolic syndrome (2013–2021), bacterial translocation (2012–2019), insulin resistance (2013–2019), t cells (2015–2021), adipose tissue (2013–2018), intestinal bacterial overgrowth (2016–2021), fatty liver disease (2013–2017), body mass index (2012–2016), leaky gut (2016–2019), hepatic stellate cells (2013–2016). The top 10 keywords in terms of burst strength include: insulin resistance (10.14), fatty liver disease (8.01), metabolic syndrome (5.33), diversity (5.05), bacterial translocation (4.89), risk factors (4.86), hepatitis b (4.76), mendelian randomisation (4.75), natural history (4.57), adipose tissue (4.44).

## Discussion

### Overview of main research results

Although research on liver cancer has reached a relatively mature stage, literature systematically exploring the connection between liver cancer and microbiota(particularly intratumoral microbiota) remains relatively limited. Furthermore, in-depth analysis of this interdisciplinary field based on bibliometric methods is even more scarce. To systematically review the development trajectory of microbiome and intratumoral microbiota in liver cancer research over the past decade, this study integrates three analytical tools—CiteSpace, VOSviewer and Bibliometrix [19] aiming to identify leading countries, core institutions, key authors and mainstream journals, further reveal research hotspots and frontier trends in this field.

Our findings indicate that, overall, the field demonstrates a sustained growth trajectory, characterized by a steady annual increase in publication output. At the national and regional level, China and the United States exert the most significant influence; notably, China not only leads in terms of publication volume but also occupies a central hub position within the international collaboration network. Among institutions, Huazhong University of Science and Technology (China) has achieved the most prolific output. The journal *Cancers* serves as the primary platform for publications on this topic. Variations in institutional rankings and collaborative projects may be attributed to technological advancements, clinical breakthroughs, and shifts in policy or funding landscapes.

### Identification of trends and future of evidence synthesis

This study systematically reveals the research hotspots and frontier trends in the field of microbiome and liver cancer from 2009 to 2025 using keyword co-occurrence network, thematic clustering and burst detection analysis [20]. From the perspective of the keyword co-occurrence network, the five major clusters formed by 129 high-frequency keywords not only reflect the multi-dimensional characteristics of research in this field but also highlight the cross-integration of core directions such as the relationship between microbiota and cancer, the gut-liver axis mechanism, and metabolic regulation [21, 22]. Among them, the cluster “Relationship between Microbiota and Cancer” contains 53 keywords, accounting for the highest proportion. Accumulating studies demonstrate that the gut microbiota is critically involved in the mechanisms, diagnosis, and therapeutic strategies of liver cancer through the gut-liver axis [23, 24]. Dysbiosis in the composition and function of the gut microbiota may influence the liver’s immune microenvironment, metabolic processes, and chronic inflammatory status, thereby contributing to the initiation and progression of hepatocellular carcinoma. This underscores the microbiota as a pivotal environmental factor in hepatocarcinogenesis, maintaining its status as a focal point of ongoing research [3, 25].

While the “Diet” cluster contains only one keyword, possibly suggesting that current exploration of dietary intervention in microbiome and liver cancer research is still in its preliminary stage, and may become a new research growth point in the future [26, 27]. Trend topic analysis shows that the top 129 keywords are all emerging fields in recent years, especially the combination of “Intratumoral Microbiota” and “Tumor Microenvironment” since 2024, which breaks through the traditional scope of gut microbiota research, reveals new mechanisms of microbiota in the local tumor microenvironment, and provides potential targets for liver cancer immunotherapy [21, 28].

The keyword burst detection results further validate the dynamic evolution of research hotspots. Long-lasting keywords such as “metabolic syndrome” and “insulin resistance” reflect the continuity of classic research directions in the association between metabolic disorders and microbiome-liver cancer [29]; While keywords with high burst intensity such as “insulin resistance” and “fatty liver disease” indicate the core position of metabolic-related mechanisms in this field, this underscores the enduring relevance of the canonical “metabolism-microbiota-inflammation-cancer” axis as the foundational framework in hepatocarcinogenesis research [30, 31]. Conversely, the nascent convergence of “intratumoral microbiota” with the “tumor microenvironment” propels the discussion toward greater granularity. It implies that future investigations should transcend the gut, focusing instead on how intratumoral microbes directly manipulate local immune suppression, alter drug responses, or function as new immunotherapeutic targets. This synergistic trajectory suggests it may evolve into a explosive research frontier in the near future [32]. Additionally, the Three field plot analysis reveals the associations between research countries, high-frequency keywords, and journals. The leadership of the United States in areas such as “immunotherapy”, the prominent contributions of China in the fields of “gut microbiome” and “hepatocellular carcinoma”, and the distinctive features of Italy in clinical intervention directions such as “probiotics” reflect the internationalization and regionalization characteristics of research in this field. The multi-level distribution of journals indicates the synergistic development of basic research and clinical translation. These results not only provide data support for understanding the current status of microbiome and liver cancer research but also offer important references for the selection of subsequent research directions.

Current research predominantly indicates correlations, yet the precise causal chains remain incompletely elucidated. Future efforts should prioritize addressing critical questions regarding the mechanisms by which specific microbial taxa or their metabolites (e.g., secondary bile acids, short-chain fatty acids) directly regulate key oncogenic signaling pathways within hepatocytes [33], as well as the spatiotemporal contributions and interactions in hepatocarcinogenesis, specifically how gut dysbiosis and intratumoral microbial colonization interplay. To overcome existing limitations, future studies should integrate emerging technological toolkits. Recommended strategies include leveraging single-cell RNA sequencing (scRNA-seq) and spatial transcriptomics to resolve cellular heterogeneity within the tumor microenvironment while simultaneously mapping microbial spatial distribution in situ, thereby unveiling “microbe–specific cell type” interaction modules [34]. Furthermore, utilizing organoid co-culture systems or humanized mouse models is essential to simulate and validate the impact of microbiota on hepatocarcinogenesis and drug responses under controlled conditions, establishing robust preclinical models [35]. Finally, developing standardized bioinformatics pipelines is crucial to tackle the analytical challenges posed by low microbial biomass and high host background noise in tumor samples, facilitating the integration and comparison of multi-center datasets [36].

### Limitations of this study

The data source is solely from WOSCC, which cannot fully encompass all articles within this field [37]. It includes only English articles and reviews, overlooking significant literature in other languages. Recent publications may not receive adequate attention due to insufficient citation counts shortly after their release. Furthermore, the keyword analysis method inherently possesses limitations; it analyzes based solely on the frequency and co-occurrence of keywords, making it challenging to fully capture the depth and complexity of research content. Emerging research directions may not be sufficiently identified due to non-standardized keyword usage or low frequency.

## Conclusion

This study systematically examines the developmental trends in the field of microbiome-intratumoral microbiota and liver cancer interactions through bibliometric analysis. The research output in this area has shown a continuous upward trend, indicating increasingly active scientific attention. Geographically, China and the United States dominate this field, with China occupying a pivotal position in the international collaboration network. Major research efforts are concentrated in institutions such as Huazhong University of Science and Technology, and a core group of authors has begun to emerge. Current research hotspots focus on the tumor microenvironment, intratumoral microbiota, metabolic-associated fatty liver disease, and the gut-liver axis, reflecting the growing recognition of the role of microbiota in immune regulation and therapeutic responses in liver cancer. Future efforts should strengthen scientific collaboration between China and the United States, as well as between China and Europe. This analysis provides important insights into the evolving trends, focal points, and future directions of the field, offering valuable references for both scholars and policymakers.

## Electronic Supplementary Material

Below is the link to the electronic supplementary material.

Supplementary Material 1

Supplementary Material 2

## Data Availability

The datasets used and analyzed during the current study are publicly available from the Web of Science Core Collection database. The data files and related documentation can be accessed at the Web of Science website (https://www.webofscience.com).
